# The PROMIS of QALYs

**DOI:** 10.1186/s12955-015-0321-6

**Published:** 2015-08-11

**Authors:** Janel Hanmer, David Feeny, Baruch Fischhoff, Ron D. Hays, Rachel Hess, Paul A. Pilkonis, Dennis A. Revicki, Mark S Roberts, Joel Tsevat, Lan Yu

**Affiliations:** Department of General Internal Medicine, University of Pittsburgh Medical Center, 230 McKee Place, Suite 600, Pittsburgh, PA 15213 USA; Department of Economics, McMaster University, Hamilton, ON, Canada; Health Utilities Incorporated, Dundas, ON Canada; Department of Engineering and Public Policy and Department of Social and Decision Sciences, Carnegie Mellon University, Pittsburgh, PA USA; Division of General Internal Medicine & Health Services Research, UCLA, Los Angeles, CA USA; Division of Health System Innovation and Research, University of Utah Schools of the Health Sciences, Salt Lake City, UT USA; Department of Psychiatry, University of Pittsburgh Medical Center, Pittsburgh, PA USA; Outcomes Research, Evidera, Bethesda, MD USA; Department of Health Policy and Management, University of Pittsburgh, Pittsburgh, PA USA; Division of General Internal Medicine, University of Cincinnati College of Medicine and Cincinnati VA Medical Center, Cincinnati, OH USA

## Abstract

Measuring health and health-related quality of life (HRQoL) is important for tracking the health of individuals and populations over time. Generic HRQoL measures allow for comparison across health conditions. One form of generic HRQoL measures are profile measures, which provide a description of health across several different domains (such as physical functioning, depression, and pain). Recent advances in health profile measurement include the development of measures based on item response theory. The Patient-Reported Outcomes Measurement Information System (PROMIS®) has been constructed using this theory. Another form of generic HRQoL measures are utility measures, which assess the value of health states. Multi-attribute utility theory provides a framework for valuing disparate domains of health and aggregating them into a single preference-based score. Such a score provides an overall measure of health outcomes as well as a quality of life weight for use in decision analyses and cost-effectiveness analyses. Developing a utility score for PROMIS® would allow simultaneous estimation of both health profile and utility scores using a single measure. The purpose of this paper is to provide a roadmap of the methodological steps necessary to create such a scoring system.

## Introduction

Patient reports about functioning and well-being, or health-related quality of life (HRQoL), are important outcomes of health care [1]. Measures of HRQoL can be targeted, providing detailed measurement about symptoms, treatment effects, and side effects. Measures can also be generic, providing an overall description of health not limited to one organ system or disease. The proliferation of HRQoL measures has made it difficult to compare results across studies that use different measures.

HRQoL can be measured using health profile measures or utility measures. Profile measures provide a description of multiple domains of health such as physical functioning, mental health, and pain [2]. These measures provide multiple scores – one for each domain of health measured.

Utility measures also cover multiple health domains but are combined into a single overall preference-based score, which is anchored on a 0 (“dead”) to 1 (“perfect health”) scale [3, 4]. This provides an overall measure of health outcomes and allows both morbidity and mortality to be captured in the form of quality-adjusted life years (QALYs). QALY measurement allows comparisons between treatment options in clinical decision-making and economic analysis.

### Recent advances in health profile measurement

Historically, HRQoL profile measures have been constructed using techniques from classical test theory that use a fixed number of questions administered to all study participants to measure an underlying construct and assume all questions are equally informative. In contrast, item response theory (IRT) [5] recognizes that questions may yield different information about the underlying construct being measured. In IRT, an underlying construct, such as physical functioning, is conceptualized as a latent trait and a large number of questions is calibrated over the entire range of the latent trait to create an item bank. Use of IRT-calibrated questions allows for any subset of questions from the bank to be used to measure an individual’s place on the continuum of the construct. The most efficient use of IRT’s flexibility is computerized adaptive testing (CAT). In a CAT administration of an IRT-based measure of physical function, for example, a respondent indicating that he or she is able to walk a mile would not be asked questions about his or her ability to walk a block. Instead, the computer selects questions about higher levels of physical functioning (such as ability to run a mile) for such a subject. This tailored testing allows more efficient and precise estimates of physical functioning with fewer questions per participant. Implementation of the PROMIS® CATs to date suggests that 4 to 6 questions are sufficient for precise estimates of health-related domains.

IRT has been used to develop a new generation of widely used health profile measures including the Patient-Reported Outcomes Measurement Information System (PROMIS®) [6]. Currently, measures for more than 44 health domains are available through the PROMIS® Assessment Center (http://www.assessmentcenter.net/) in the form of item banks, short forms, and CAT administration. The breadth and precision of the PROMIS® measures can be selected according to the needs of each study.

### Benefits of combining irt health profile measurement and utility measurement

The EQ-5D, Health Utilities Index, SF-6D, and the Quality of Well-being Index [7] are among the most widely used generic preference-based measures. Like questionnaires developed using classical test theory, they each have some of the following limitations: (1) large proportions of the respondents scoring at the very top or very bottom of the scale in some populations of interest (i.e., ceiling effects in the very healthy or floor effects in the very ill), (2) imprecise measurement for individuals, (3) poorly-worded questions such as those that combine two concepts (double-barreled questions), and (4) differences in range of domains covered [7]. While modification of a particular instrument may overcome some of these problems, modification also results in concerns about comparability of results obtained with different versions of the same instrument.

PROMIS® provides an opportunity to address several limitations of the existing generic preference measures including: (1) fully capturing the entire range of a construct, (2) measuring an individual’s health status with greater precision, and (3) creating a standardized valuation methodology for future studies. The PROMIS® measures stand to be highly applicable across clinical, research, and population studies. Thus, creating a utility scoring system for PROMIS® would allow efficient use of study resources to collect both health profile and health utility scores.

### Proposed solution

Various methods could be used to derive preference-based scores for PROMIS®. Using methods such as regression analysis, one could predict scores on an existing index such as the EQ-5D from PROMIS® scores [8] but this method is limited by the reliability of the measures being used and their degree of overlap [9]. Another method is to use a subset of PROMIS® (such as the PROMIS® profile) as if it is a static questionnaire and construct a preference-based scoring system for it using the same techniques as legacy utility measures [10]. Neither method takes advantage of characteristics of latent constructs to ensure that the full range of health is measured. Also, neither method takes advantage of IRT to improve the precision of measurement.

Instead, we propose developing a health utility measure that builds on the advantages of the IRT-based measures such as PROMIS® and can be estimated from them. Our proposed method will allow both the granularity of individual health domains and the simplicity of a single overall utility score to be calculated from the same data. This method creates a domain-specific utility function over each construct. Once the relationship between utility and a particular construct is established, the utility weight for a specific score on the construct can be estimated. A set of utility weights for a set of constructs can then be combined into a single utility score by using multi-attribute utility theory (MAUT).

Implementation of this approach requires four distinct steps:

1. * Selecting key domains for valuation*. MAUT works best when the selected domains are structurally independent of each other. This means that the range of possible outcomes on one domain is not logically limited by the outcome on the other domain (and vice versa). For example, physical function and depression are structurally independent because one can imagine an individual with excellent physical function and very severe depression; one can also imagine an individual who is not depressed but has very poor physical function. Structural independence is conceptual rather than epidemiological; domains may be conceptually independent even if they are highly correlated in real-life samples. Further, for ease of valuation and use, the set of key domains should be parsimonious.
2. * Developing a method to value an individual health domain*. If a single question with multiple response options (e.g., *never, rarely, sometimes, often, almost always*) spans the entire range of the construct and is considered to have good conceptual representation of it, then that question could be used to construct the utility function for that domain. Finding such an item in item banks is unusual, so empirical work should assess the feasibility and comparability of methods such as selecting and combining two questions that span the construct or constructing health-state descriptions to represent intervals along the construct [11]. This step should produce a function that can assign a utility value for any level of functioning within the domain.
3. * Comparing the relative weight of each health domain in determining the overall score by using corner states for the parsimonious set of domains selected in step 1*. A corner state is a multi-domain health state description where one domain is at one extreme (the worst level of function) and all other domains are at the other extreme (the best level of function).
4. * Combining the single attribute utility function for individual domains with their weights relative to all other domains to create an overall utility score*. One can use MAUT methods to derive an algorithm for combining the single attribute utility functions for each domain.

A societal scoring function, like those provided by the EQ-5D or Health Utilities Index, will require that these preference elicitation steps be performed using a representative sample of a population of interest (such as a country). To make such a scoring function useful, it needs to be applied in a variety of samples to provide context and guidelines for interpretation. The alternative to address the known issues in the current health utility measures – developing another health utility measure using the same techniques as the legacy measures – would add to an already crowded field, is unlikely to improve upon the known issues without causing other problems like increased response burden,  and would not have the benefit of experience that the legacy measures enjoy.

## Conclusion

As delineated above, a variety of methodological and empirical advances still need to be accomplished to demonstrate feasibility of this approach before constructing a scoring function from the general population. We have begun undertaking such a project based on PROMIS®, as these advances have the potential to increase substantially the information patients, clinicians, researchers, and policy makers need to make healthcare decisions. The resultant preference-based instrument would allow for precise description and valuation of health outcomes in clinical trials, clinical care, and the general population.

## Abbreviations

PROMIS®: Patient-reported outcomes measurement information system
IRT: Item response theory
CAT: Computerized adaptive testing
HRQoL: Health-related quality of life
SF-6D: Short form 6 dimensions
EQ-5D: EuroQol 5 dimensions
MAUT: Multi-attribute utility theory
